# COVID-19 Vaccine Uptake, Acceptance, and Hesitancy Among Persons With Mental Disorders During the Second Stage of China's Nationwide Vaccine Rollout

**DOI:** 10.3389/fmed.2021.761601

**Published:** 2021-11-11

**Authors:** Hui Huang, Xiao-Min Zhu, Peng-Wei Liang, Zhong-Ming Fang, Wei Luo, Yi-Ming Ma, Bao-Liang Zhong, Helen Fung-Kum Chiu

**Affiliations:** ^1^Affliated Wuhan Mental Health Center, Tongji Medical College of Huazhong University of Science and Technology, Wuhan, China; ^2^Department of Psychiatry, Suzhou Guangji Hospital, The Affiliated Guangji Hospital of Soochow University, Suzhou, China; ^3^Faculty of Psychology, Beijing Normal University, Beijing, China; ^4^Research Center for Psychological and Health Sciences, China University of Geosciences, Wuhan, China; ^5^Department of Psychiatry, The Chinese University of Hong Kong, Hong Kong SAR, China

**Keywords:** COVID-19, vaccine, uptake, acceptance, hesitancy, mental disorders, China

## Abstract

Persons with mental disorders (PwMDs) are a priority group for COVID-19 vaccination, but empirical data on PwMDs' vaccine uptake and attitudes toward COVID-19 vaccines are lacking. This study examined the uptake, acceptance, and hesitancy associated with COVID-19 vaccines among Chinese PwMDs during China's nationwide vaccine rollout. In total, 906 adult PwMDs were consecutively recruited from a large psychiatric hospital in Wuhan, China, and administered a self-report questionnaire, which comprised standardized questions regarding sociodemographics, COVID-19 vaccination status, attitudes toward COVID-19 vaccines, and psychopathology. Vaccine-recipients were additionally asked to report adverse events that occurred following vaccination. PwMDs had a much lower rate of vaccination than Wuhan residents (10.8 vs. 40.0%). The rates of vaccine acceptance and hesitancy were 58.1 and 31.1%, respectively. Factors associated with vaccine uptake included having other mental disorders [odds ratio (OR) = 3.63], believing that ≥50% of vaccine-recipients would be immune to COVID-19 (OR = 3.27), being not worried about the side effects (OR = 2.59), and being an outpatient (OR = 2.24). Factors associated with vaccine acceptance included perceiving a good preventive effect of vaccines (OR = 12.92), believing that vaccines are safe (OR = 4.08), believing that ≥50% of vaccine-recipients would be immune to COVID-19 (OR = 2.20), and good insight into the mental illness (OR = 1.71). Adverse events occurred in 21.4% of vaccine-recipients and exacerbated pre-existing psychiatric symptoms in 2.0% of vaccine-recipients. Nevertheless, 95.2% of vaccine-recipients rated adverse events as acceptable. Compared to the 58.1% vaccine acceptance rate and the 40.0% vaccination rate in the general population, the 10.8% vaccine coverage rate suggested a large unmet need for COVID-19 vaccination in Chinese PwMDs. Strategies to increase vaccination coverage among PwMDs may include provision of reliable sources of information on vaccines, health education to foster positive attitudes toward vaccines, a practical guideline to facilitate clinical decision-making for vaccination, and the involvement of psychiatrists in vaccine consultation and post-vaccination follow-up services.

## Introduction

During the ongoing COVID-19 pandemic, accumulating evidence has shown significant associations of pre-existing mental disorders with an increased risk of COVID-19 infection and COVID-19-related physical complications and mortality (1–3). Accordingly, there have been increasing calls for prioritizing persons with mental disorders (PwMDs) for COVID-19 vaccination, and a few countries have prioritized PwMDs in their updated vaccination strategies (4–6). Findings from acceptability studies of COVID-19 vaccines have revealed that the intention to get vaccinated against COVID-19 is associated with an individual's psychological characteristics (7, 8). Due to the impaired insight and decision-making capacity of PwMDs, caution is needed when seeking informed consent from these individuals for COVID-19 vaccination (9). To maximize the uptake of COVID-19 vaccines among PwMDs, more targeted interventions are warranted. Importantly, empirical data on PwMDs' attitudes toward COVID-19 vaccination are a prerequisite for developing an effective vaccination strategy.

To date, the acceptance of and hesitancy toward COVID-19 vaccines have been extensively examined in general populations of various countries (7, 8, 10–16). In these studies, 37.3–83.6% of the adults were willing to receive the vaccine, while 17.1–62.6% of them were hesitant (11, 16). Commonly reported factors associated with acceptance included male sex, old age, fear of COVID-19 infection, and trust in the efficacy and safety of COVID-19 vaccines; while factors associated with hesitancy included concerns about the efficacy and side effects of COVID-19 vaccines, not having received the influenza vaccine, and no trustworthy information sources related to COVID-19 vaccines (11, 12, 14, 17, 18). However, available data regarding PwMDs' intention to get vaccinated and uptake of the COVID-19 vaccine have been very limited. To our knowledge, only one empirical study from Denmark has investigated the acceptability of COVID-19 vaccines among PwMDs (19). This study found an 84.8% acceptance rate of COVID-19 vaccines among PwMDs, which was slightly lower than that in the Danish general population (89.5%). Nevertheless, because considerable variations in COVID-19 vaccine acceptance rates in general populations have been observed across countries (10), it remains unknown whether PwMDs have similarly high acceptance rates in other countries. In addition, the relationships between vaccine acceptance and vaccination and psychiatric symptoms among PwMDs may inform the development of a focused vaccination strategy. Unfortunately, the aforementioned study did not focus on this topic.

A notable limitation of prior studies on COVID-19 vaccine acceptance is that almost all of them were conducted before COVID-19 vaccines were available to the public (20). Because determinants of vaccine acceptance are context-dependent and attitudes and beliefs toward vaccines are different between persons with and without mental disorders (20, 21), it is difficult to generalize the findings from prior studies to PwMDs during the recent mass rollout of COVID-19 vaccines. Furthermore, given the large gap between the intention and actual behavior to receive a vaccine against COVID-19 (20, 21), it is important to additionally examine the uptake of COVID-19 vaccines, but few studies have examined this topic. As suggested by an influenza vaccination study in the United States, the uptake rate of influenza vaccine is much lower in PwMDs than in the general population (28.4 vs. 40.9%) (22).

China's current nationwide COVID-19 vaccination program was implemented in a two-stage manner. The first stage, from December 2020 to January 2021, focused on nine subpopulations with a high risk for COVID-19 infection, including medical staff, inspection and quarantine personnel, international migrant workers, and cold-chain food workers (15). The second stage focused on adult residents and was launched since February 2021, a period when COVID-19 vaccines had been widely available for Chinese residents (23). To increase the accessibility of vaccine administration services, vaccines are freely provided to all residents by the Chinese government, and all community-dwelling residents could conveniently take vaccines at primary care facilities nearest to their residence places. Nevertheless, in China, PwMDs are not listed in the priority subpopulations for COVID-19 vaccines (9). Moreover, there are also no clinical guidelines for the vaccination in PwMDs (23, 24), which may result in difficulties in clinical decision-making regarding COVID-19 vaccination in this population. For example, since the nationwide COVID-19 vaccination program, in our hospital, an increasing number of psychiatric patients have sought clarifications on their eligibility to receive the vaccine, but no clear answers were provided.

In China, some debates exist as to whether PwMDs should be prioritized to take COVID-19 vaccines (9). Given the importance of patient involvement in clinical decision-making process (25), understanding PwMDs' attitudes toward COVID-19 vaccines would facilitate the decision making for vaccination in this vulnerable population. In addition, first-hand data on vaccination status among PwMDs and vaccinated PwMDs' post-vaccination experiences are helpful for developing an effective vaccination strategy in this population. Research questions to be answered in this study are (i) How many psychiatric patients have got vaccinated and how many PwMDs are willing to take the vaccines? (ii) What factors are associated with uptake and acceptance of the vaccines? and (iii) What are the subjective experiences of PwMDs after getting vaccinated? This study examined acceptance, hesitancy, and uptake of COVID-19 vaccines in Chinese PwMDs during the second stage of China's nationwide vaccine rollout, as well as the subjective experiences of vaccine-recipients among PwMDs.

## Methods

### Subjects and Settings

Between March 24 and April 27, 2021, a cross-sectional survey was conducted at Wuhan Mental Health Center, which is the largest psychiatric specialty hospital in Wuhan, China. The center has 950 inpatient beds and mainly provides mental health services to local residents, with catchment areas of ~ 10 million people. The average annual total number of outpatient visits and hospital admissions were 330,000 and 11,000, respectively, in the past 5 years. We consecutively enrolled both outpatients and inpatients who were 18 years old or older, had confirmed diagnoses of mental disorders, sought psychiatric treatment at the center during the survey period, and were deemed “able to complete the survey” by their treating psychiatrists, and voluntarily participated in the study. We excluded patients who had been infected with COVID-19 and those who were considered “unable to complete the survey” due to severe physical illnesses or cognitive disorders by their treating psychiatrists. According to an official report, the total number of COVID-19 vaccination doses administered in Wuhan had reached ~4 million by April 7, 2021 (26), translating to a vaccine uptake rate of roughly 40.0% in Wuhan residents during the period of our survey.

Vaccine acceptance was the primary outcome of interest of this study. In our pilot study, the prevalence of vaccine acceptance was 52.9%. Therefore, parameters needed for estimating the sample size of this cross-sectional study were set as below (27): (1) a prevalence of 0.53, (2) a confidence interval of 95%, (3) a confidence interval width of 0.07, and (4) a response rate of 0.90. By using PASS 11 (LLC, Kaysville, UT, USA), the minimum sample size of PwMDs was estimated to be 898.

The authors assert that all procedures contributing to this work complied with the ethical standards of the relevant national and institutional committees on human experimentation and with the Helsinki Declaration of 1975, as revised in 2008. The study protocol was approved by the Institutional Review Board of Wuhan Mental Health Center. Informed consent was obtained from the participating patients or their guardians when necessary.

### Procedures and Measures

All participants completed a self-administered anonymous questionnaire, which was provided in an online or paper-pencil manner, depending on participants' preference. Six trained psychiatrists and master students in clinical psychology were assigned to recruit participants, facilitate the completion of the questionnaire, and check the quality of the questionnaire before submission. These investigators also read out questions for participants who had difficulties in completing the questionnaire. Before the main study, a pilot study (*n* = 17) was conducted to test the feasibility of study procedures and questionnaire, and the survey questionnaire was finalized thereafter.

Sociodemographic variables in the questionnaire included sex, age, education, marital status, and self-rated family financial status (good, fair, poor).

We used a checklist to assess the presence of chronic medical conditions (28), including heart disease, hypertension, stroke and other cerebrovascular diseases, diabetes, chronic obstructive pulmonary disease, cancer, chronic nephritis, and chronic hepatitis.

Risk perception of COVID-19 (Table 1). Five questions were used to assess participants' perceived likelihood of COVID-19 infection, risk of death due to COVID-19 if a person were infected, infectivity of COVID-19, and level of agreement on large-scale secondary outbreaks of COVID-19 in China.

**Table 1:** Questions used for assessing risk perception of COVID-19, negative impact of the COVID-19 pandemic, COVID-19 vaccination, and attitudes toward COVID-19 vaccines in the survey questionnaire.

**Variables**	**Questions**	**Response option**
**Risk perception of COVID-19**		
Likelihood of COVID-19 infection	Would you please give me an estimate of the likelihood that you would be infected with COVID-19?	Very high, high, low, very low
Risk of death due to COVID-19 infection	To the best of your knowledge, what is the possibility of dying due to COVID-19 if a person were infected with COVID-19?	Very high, high, low, very low, unknown
Infectivity of COVID-19	To the best of your knowledge, what is the infectivity of COVID-19?	Very high, high, low, very low
Re-outbreak of COVID-19	Do you agree that there would be another large-scale COVID-19 outbreak in the future in China?	Very agree, agree, disagree, very disagree
**Negative impact of the COVID-19 pandemic**		
Economic income	What is the impact of the COVID-19 pandemic on your income?	No impact, decrease, substantial decrease
Daily life	What is the impact of the COVID-19 pandemic on your current daily life?	No impact, inconvenience, substantial inconvenience
**COVID-19 vaccination**		
General attitude toward COVID-19 vaccination	Do you agree that persons with mental disorders should take COVID-19 vaccines?”	Agree, disagree, unknown
Vaccine acceptance and hesitancy	Are you willing to take a COVID-19 vaccine?	Yes, no, unsure
Vaccine uptake	Have you taken the COVID-19 vaccine?	Yes, no
**Attitudes toward COVID-19 vaccines**		
Preventative effect of COVID-19 vaccines	To what extent do you agree that COVID-19 vaccines are effective in preventing COVID-19?”	Very agree, agree, disagree, very disagree
% of immunized persons among vaccine-recipients	To the best of your knowledge, how many people would be immunized to COVID-19 after getting vaccines against COVID-19?	All, most, a half, some, none
Safety of COVID-19 vaccines	To the best of your knowledge, how safe is the COVID-19 vaccine?	Very safe, safe, unsafe, very unsafe
Worry about side effects of COVID-19 vaccines	Are you worried about side effects of the COVID-19 vaccine?	Very worried, worried, not worried
**Subjective experiences of vaccine-recipients**		
Side effects	Do you have any of the following side effects after taking the COVID-19 vaccine?	None, deterioration of pre-existing psychiatric symptoms, local adverse reactions (i.e., injection site pain, itching, induration, redness, swelling), fatigue, fever, muscle pain, headache, cough, diarrhea, nausea, anorexia, allergy, others (please indicate______)
Acceptability of side effect	Do you think that the above reported side effects are acceptable?	Acceptable, unacceptable

Two questions were used to assess the negative impact of the COVID-19 pandemic on the economic income and current daily life of PwMDs (Table 1).

COVID-19 vaccination and attitudes toward COVID-19 vaccines (Table 1). Participants were asked whether they agreed that PwMDs should take COVID-19 vaccines, whether they had taken the COVID-19 vaccine, and whether they were willing to take a COVID-19 vaccine. In accordance with previous studies, persons who were willing to take the vaccine were vaccine-accepting individuals, while persons who were unwilling or unsure about taking the vaccine were vaccine-hesitant individuals (18, 29). Other attitude questions included the perceived preventive effect of COVID-19 vaccines, perceived percentage of vaccine-recipients who would be immunized to COVID-19, perceived safety of COVID-19 vaccines, and worry about side effects of COVID-19 vaccines.

#### Clinical Characteristics

Clinical variables included clinical setting (outpatient vs. inpatient), primary diagnosis of mental disorder, family history of mental disorders, insight into mental disorders, and psychiatric symptoms. Two items adapted from the Chinese Insight and Treatment Attitudes Questionnaire (30), were used to assess patients' insight into mental disorders: “Do you agree that you have a mental health problem?” and “Do you agree that you are in need of psychiatric treatment to manage your mental health problem or maintain your mental health?” Each question was rated on a three-point scale: 1 = completely agree, 2 = partly agree, and 3 = disagree. Total scores of the two insight items of two, three to five, and six were operationally defined as good, partial, and poor insight, respectively. Depressive symptoms were assessed with the Chinese two-item version of Patient Health Questionnaire (PHQ-2) (31). In China, a cutoff score of two or more is used to denote the presence of clinically significant depressive symptoms (32). Insomnia symptoms were assessed by asking the following question: “In the past month, how often do you have difficulties falling asleep, maintaining sleep, or waking too early and getting back to sleep?” (33). Responses were coded as no, occasionally, sometimes, often, and always. Respondents answering “often” and “always” to the question were classified as having insomnia symptoms.

Subjective experiences of COVID-19 vaccine-recipients. Vaccine-recipients were additionally asked to report side effects that occurred after receiving the COVID-19 vaccine, in particular the exacerbation of pre-existing psychiatric symptoms and acceptability of side effects (Table 1).

### Statistical Analysis

Prevalence rates of vaccination and vaccine acceptance and hesitancy were calculated. Chi-square test was used to compare sociodemographic characteristics, risk perception of COVID-19, perceived negative impact of the COVID-19 pandemic, attitudes toward COVID-19 vaccines, and clinical characteristics between vaccine-recipients and vaccine-hesitant individuals. The Multiple logistic regression model with a backward stepwise entry of all significant variables in the Chi-square test was used to identify factors associated with the vaccine uptake. Specifically, the backward stepwise model started with all significant variables from the univariate analysis, and then removed the variable with the largest P value. Next, the model was refitted and the variable with the largest *P*-value in the new model was removed. This process was repeated to eliminate variables one-by-one until no further variables can be deleted without a statistically insignificant loss of fit (34). Factors associated with vaccine acceptance were identified in the same manner. The subjective experiences of vaccine-recipients were presented as frequencies and percentages. Odds ratios (ORs) and their 95% confidence intervals (95% CIs) were used to quantify associations between factors and outcomes. The statistical significance level was set at *P* < 0.05 (two-sided). SPSS software version 18.0 package (SPSS Inc., Chicago, IL, USA) was used for all analyses.

## Results

Our analysis included a final sample of 906 psychiatric patients: 317 outpatients (35.0%) and 589 inpatients (65.0%). The average age of this sample was 36.2 years (standard deviation [SD]: 13.2, range: 18-78) and 355 (39.2%) were men. Detailed sociodemographic, COVID-19-related, and clinical characteristics of the total sample are displayed in the third column of Table 2.

**Table 2:** Characteristics of COVID-19 vaccine-recipients and vaccine-accepting individuals in comparison to vaccine-hesitant individuals among Chinese persons with mental disorders (PwMDs), *n* (%).

**Variables**		**Total sample (*n* = 906)**	**Vaccine-recipients (*n* = 98)**	**Vaccine-accepting individuals (*n* = 526)**	**Vaccine-hesitant individuals (*n* = 282)**	**Take vs. hesitancy**		**Acceptance vs. hesitancy**	
						**χ2**	***P***	**χ2**	***P***
**Sociodemographic**									
Sex	Male	355 (39.2)	31 (31.6)	217 (41.3)	107 (37.9)	1.252	0.263	0.838	0.360
	Female	551 (60.8)	67 (68.4)	309 (58.7)	175 (62.1)				
Age group (years)	18-34	477 (52.6)	49 (50.0)	291 (55.3)	137 (48.6)	3.827	0.148	6.642	0.036
	35-44	286 (31.6)	37 (37.8)	162 (30.8)	87 (30.9)				
	45+	143 (15.8)	12 (12.2)	73 (13.9)	58 (20.6)				
Education	Middle school and below	498 (55.0)	39 (39.8)	288 (54.8)	171 (60.6)	12.779	<0.001	2.592	0.107
	College or above	408 (45.0)	59 (60.2)	238 (45.2)	111 (39.4)				
Marital status	Never-married	464 (51.2)	40 (40.8)	281 (53.4)	143 (50.7)	8.713	0.013	1.195	0.550
	Married, remarried, or cohabiting	330 (36.4)	50 (51.0)	182 (34.6)	98 (34.8)				
	Divorced, separated, or widowed	112 (12.4)	8 (8.2)	63 (12.0)	41 (14.5)				
Family financial status	Good	152 (16.8)	27 (27.6)	79 (15.0)	46 (16.3)	7.935	0.019	1.575	0.455
	Fair	598 (66.0)	60 (61.2)	358 (68.1)	180 (63.8)				
	Poor	156 (17.2)	11 (11.2)	89 (16.9)	56 (19.9)				
Chronic medical condition	No	695 (76.7)	81 (82.7)	403 (76.6)	211 (74.8)	2.506	0.113	0.324	0.569
	Yes	211 (23.3)	17 (17.3)	123 (23.4)	71 (25.2)				
**Risk perception of COVID-19**									
Likelihood of COVID-19 infection	Low	816 (90.1)	88 (89.8)	468 (89.0)	260 (92.2)	0.544	0.461	2.141	0.143
	High	90 (9.9)	10 (10.2)	58 (11.0)	22 (7.8)				
Risk of death due to COVID-19 infection	Low	282 (31.1)	37 (37.8)	145 (27.6)	100 (35.5)	3.971	0.137	16.387	<0.001
	High	476 (52.5)	49 (50.0)	305 (58.0)	122 (43.3)				
	Unknown	148 (16.3)	12 (12.2)	76 (14.4)	60 (21.3)				
Infectivity of COVID-19	Low	107 (11.8)	12 (12.2)	50 (9.5)	45 (16.0)	0.786	0.375	7.365	0.007
	High	799 (88.2)	86 (87.8)	476 (90.5)	237 (84.0)				
Large-scale re-outbreak of COVID-19	Disagree	728 (80.4)	75 (76.5)	422 (80.2)	231 (81.9)	1.345	0.246	0.337	0.562
	Agree	178 (19.6)	23 (23.5)	104 (19.8)	51 (18.1)				
**Negative impact of the COVID-19 pandemic**									
Economic income	No impact	445 (49.1)	52 (53.1)	234 (44.5)	159 (56.4)	0.325	0.569	10.400	0.001
	Reduction	461 (50.9)	46 (46.9)	292 (55.5)	123 (43.6)				
Daily life	No impact	324 (35.8)	39 (39.8)	174 (33.1)	111 (39.4)	0.006	0.940	3.173	0.075
	Inconvenience	582 (64.2)	59 (60.2)	352 (66.9)	171 (60.6)				
**Attitudes toward COVID-19 vaccines**									
COVID-19 vaccination in PwMDs	Agree	561 (61.9)	79 (80.6)	411 (78.1)	71 (25.2)	93.617	<0.001	218.224	<0.001
	Disagree	76 (8.4)	4 (4.1)	18 (3.4)	54 (19.1)				
	Unknown	269 (29.7)	15 (15.3)	97 (18.4)	157 (55.7)				
Preventive effect of vaccines	Good	852 (94.0)	94 (95.9)	522 (99.2)	236 (83.7)	9.520	0.002	76.482	<0.001
	Bad	54 (6.0)	4 (4.1)	4 (0.8)	46 (16.3)				
% of immunized persons among vaccine-recipients	≥50%	842 (92.9)	94 (95.9)	509 (96.8)	239 (84.8)	8.367	0.004	38.560	<0.001
	<50%	64 (7.1)	4 (4.1)	17 (3.2)	43 (15.2)				
Safety of vaccines	Safe	854 (94.3)	94 (95.9)	518 (98.5)	242 (85.8)	7.250	0.007	52.686	<0.001
	Unsafe	52 (5.7)	4 (4.1)	8 (1.5)	40 (14.2)				
Worry about the side effects of vaccines	Worried	568 (62.7)	48 (49.0)	315 (59.9)	205 (72.7)	18.382	<0.001	13.131	<0.001
	Not worried	338 (33.3)	50 (51.0)	211 (40.1)	77 (27.3)				
**Clinical**									
Setting	Outpatient	317 (35.0)	54 (55.1)	164 (31.2)	99 (35.1)	12.090	0.001	1.290	0.256
	Inpatient	589 (65.0)	44 (44.9)	362 (68.8)	183 (64.9)				
Family history of mental disorder	Yes	198 (21.9)	19 (19.4)	121 (23.0)	58 (20.6)	0.063	0.802	0.632	0.427
	No	708 (78.1)	79 (80.6)	405 (77.0)	224 (79.4)				
Diagnosis	Psychotic disorders	231 (25.5)	10 (10.2)	149 (28.3)	72 (25.5)	20.995	<0.001	5.628	0.131
	Mood disorders	316 (34.9)	23 (23.5)	198 (37.6)	95 (33.7)				
	Anxiety disorders	117 (12.9)	16 (16.3)	67 (12.7)	34 (12.1)				
	Other disorders	242 (26.7)	49 (50.0)	112 (21.3)	81 (28.7)				
Insight into mental disorders	Poor	186 (20.5)	33 (33.7)	86 (16.3)	67 (23.8)	3.930	0.140	8.024	0.018
	Partial	455 (50.2)	41 (41.8)	271 (51.5)	143 (50.7)				
	Good	265 (29.2)	24 (24.5)	169 (32.1)	72 (25.5)				
Depressive symptoms	No	360 (39.7)	54 (55.1)	196 (37.3)	110 (39.0)	7.680	0.006	0.238	0.626
	Yes	546 (60.3)	44 (44.9)	330 (62.7)	172 (61.0)				
Insomnia symptoms	No	682 (75.3)	78 (79.6)	401 (76.2)	203 (72.0)	2.184	0.139	1.757	0.185
	Yes	224 (24.7)	20 (20.4)	125 (23.8)	79 (28.0)				

In total, 561 patients (61.9%) agreed that PwMDs should take COVID-19 vaccines, 98 (10.8%) had taken the vaccine at the time of this survey, 526 (58.1%) reported that they were willing to take the vaccine, and 282 (31.1%) were hesitant to take the vaccine (17.1% unwilling and 14.0% unsure).

Compared to vaccine-hesitant persons, vaccine-recipients were more likely to have a college-level education or above (60.2 vs. 39.4%, *P* < 0.001), be married, remarried, or cohabiting (51.0 vs. 34.8%, *P* = 0.013), rate their family financial status as “good” (27.6 vs. 16.3%, *P* = 0.019), agree that the preventive effect of vaccines is good (95.9 vs. 83.7%, *P* = 0.002), believe that at least half of vaccine-recipients would be immune to COVID-19 (95.9 vs. 84.8%, *P* = 0.004), believe that vaccines are safe (95.9 vs. 85.8%, *P* = 0.007), be not worried about the side effects of vaccines (51.0 vs. 27.3%, *P* < 0.001), be outpatients (55.1 vs. 35.1%, *P* = 0.001), have mental disorders other than psychotic, mood, and anxiety disorders (50.0 vs. 28.7%, *P* < 0.001), and be not depressed (55.1 vs. 39.0%, *P* = 0.006) (Table 2). In the multiple logistic regression, factors significantly associated with vaccine uptake were an educational attainment of college or above (OR = 1.99, *P* = 0.010), a good family financial status (OR = 2.76, *P* = 0.027), believing that ≥50% of vaccine-recipients would be immune to COVID-19 (OR = 3.27, *P* = 0.034), being not worried about the side effects of vaccines (OR = 2.59, *P* < 0.001), being outpatients (OR = 2.24, *P* = 0.003), and having other mental disorders (OR = 3.63, *P* = 0.001) (Table 3).

**Table 3:** Factors associated with COVID-19 vaccine uptake and acceptance among Chinese persons with mental disorders (reference category: vaccine hesitancy).

**Variables**		**OR (95%CI)**	***P***
**Vaccine uptake**			
Education	Middle school or below	1	
	College or above	1.99 (1.18, 3.35)	0.010
Family financial status	Poor	1	
	Good	2.76 (1.13, 6.79)	0.027
% of immunized persons among vaccine-recipients	<50% ≥50%	1 3.27 (1.10, 9.73)	0.034
Worry about the side effects of vaccines	Worried	1	
	Not worried	2.59 (1.53, 4.39)	<0.001
Setting	Inpatient	1	
	Outpatient	2.24 (1.33, 3.79)	0.003
Diagnosis	Psychotic disorders	1	
	Other disorders	3.63 (1.65, 8.01)	0.001
**Vaccine acceptance**			
Risk of death due to COVID-19 infection	Low	1	
	High	1.64 (1.14, 2.36)	0.007
Negative impact of the COVID-19 pandemic on economic income	No impact Reduced	1 1.68 (1.22, 2.31)	0.002
Preventive effect of domestic vaccines	Bad	1	
	Good	12.92 (4.41, 37.85)	<0.001
% of immunized persons among vaccine-recipients	<50% ≥50%	1 2.20 (1.08, 4.45)	0.029
Safety of domestic vaccines	Unsafe	1	
	Safe	4.08 (1.72, 9.65)	0.001
Insight into mental disorder	Poor	1	
	Good	1.71 (1.07, 2.73)	0.024

Compared to vaccine-hesitant persons, vaccine-accepting individuals were more likely to be aged 18-34 years (55.3 vs. 48.6%, *P* = 0.036), perceive a high risk of death due to COVID-19 infection (58.0 vs. 43.3%, *P* < 0.001), rate the infectivity of COVID-19 as high (90.5 vs. 84.0%, *P* = 0.007), have reduced income due to the COVID-19 pandemic (55.5 vs. 43.6%, *P* = 0.001), agree that the preventive effect of vaccines is good (99.2 vs. 83.7%, *P* < 0.001), believe that ≥50% of the vaccine-recipients would be immune to COVID-19 (96.8 vs. 84.8%, *P* < 0.001), believe that vaccines are safe (98.5 vs. 85.8%, *P* < 0.001), be not worried about the side effects of vaccines (40.1 vs. 27.3%, *P* < 0.001), and have good insight into mental disorders (32.1 vs. 25.5%, *P* = 0.018) (Table 2). In the multiple logistic regression, factors significantly associated with vaccine acceptance were perceiving a high risk of death due to COVID-19 infection (OR = 1.64, *P* = 0.007), having reduced income due to the COVID-19 pandemic (OR = 1.68, *P* = 0.002), perceiving a good preventive effect of vaccines (OR = 12.92, *P* < 0.001), believing that ≥50% of vaccine-recipients would be immune to COVID-19 (OR = 2.20, *P* = 0.029), believing that vaccines are safe (OR = 4.08, *P* = 0.001), and having good insight into mental disorders (OR = 1.71, *P* = 0.024)

Among the 98 vaccine-recipients, 21 (21.4%) reported at least one adverse event (Table 4); of whom 20 (95.2%) believed these side effects were acceptable while only one (4.8%) felt unacceptable because of muscle pain. The most common adverse event was local adverse reactions (*n* = 9, 9.2%), followed by muscle pain (*n* = 7, 7.1%). Two patients (2.0%) endorsed exacerbated psychiatric symptoms after the vaccine uptake (Table 4).

**Table 4:** Adverse events after getting the COVID-19 vaccine among the 98 COVID-19 vaccine-recipients.

**Adverse event**	***n* (%)**
None	77 (78.6)
Any adverse event	21 (21.4)
Deterioration of pre-existing psychiatric symptoms	2 (2.0)
Local adverse reactions	9 (9.2)
Fatigue	2 (2.0)
Fever	1 (1.0)
Muscle pain	7 (7.1)
Headache	2 (2.0)
Cough	0 (0.0)
Diarrhea	1 (1.0)
Nausea	2 (2.0)
Anorexia	0 (0.0)
Allergy	0 (0.0)
Others	1 (0.0)

## Discussion

To the best of our knowledge, this is the first study in China that examined the uptake and acceptance of COVID-19 vaccines among PwMDs during the second stage of China's nationwide vaccine rollout. The main findings of this study are: first, the vaccination rate among PwMDs was 10.8%, which was much lower than the 40.0% concurrent vaccination rate among Wuhan residents; second, the rates of vaccine acceptance and hesitancy among PwMDs were 58.1 and 31.1%, respectively; third, in addition to sociodemographic variables, COVID-19-related and clinical factors were associated with vaccine uptake and acceptance; and, fourth, although adverse events occurred in 21.4% of the vaccine-recipients, the vast majority (95.2%) of PwMDs reported that the adverse events they experienced were acceptable. Notably, 2.0% of the vaccine-recipients reported exacerbated pre-existing psychiatric symptoms.

In the Chinese general population, rates of vaccine acceptance and hesitancy were 67.1-88.6 and 11.4-32.9%, respectively (10, 15). Compared to the general population, PwMDs have a slightly lower level of acceptance and a relatively higher level of hesitancy, suggesting that PwMDs are less willing to get vaccinated. Nevertheless, the relatively low acceptance rate should not be the primary reason for the very low vaccine coverage rate among PwMDs. Jefsen and colleagues have argued that vaccine hesitancy is not a major barrier for vaccine uptake among PwMDs in Denmark (19). Accordingly, the present study revealed a very large gap between vaccine acceptance and uptake rates (58.0 vs. 10.8%) in Chinese PwMDs. We speculate that other barriers to vaccination that are specific to mental disorders in China may complicate vaccine uptake such as controversy regarding the priority of PwMDs for COVID-19 vaccination, stigma surrounding mental disorders, impaired decision-making ability of PwMDs, and lack of clinical guidelines for vaccination for PwMDs.

In prior studies, a high level of education, major medical conditions, and trust in the efficacy, safety, and benefits of the COVID-19 vaccine were significant factors associated with vaccine uptake in the general population (15, 20). Similarly, in the present study, having a college-level education or above, believing that ≥50% of vaccine-recipients would be immune to COVID-19, and being not worried about the side effects of the vaccine were significant factors associated with vaccine uptake in PwMDs. Since vaccines are provided free of charge in China, the significant association between good family economic status and vaccine uptake should not be ascribed to the higher vaccine affordability for economically advantageous PwMDs. We speculate that PwMDs with a good economic status might have less burden of childcare and be more likely to use smartphones to successfully make appointments for COVID-19 vaccination.

Among PwMDs, we did not replicate significant relationships of vaccine acceptance with sociodemographic factors that have been reported in the general population (11, 12, 14, 17, 18). Instead, only COVID-19-related factors were associated with vaccine acceptance such as risk perception of COVID-19 and perceived efficacy and safety of the COVID-19 vaccine, which are partly consistent with findings from general population-based studies (11, 12, 14, 15, 17, 18, 20). These findings are interesting given that knowledge and attitudes toward COVID-19 and vaccines are modifiable and can be improved via health education (35).

The unique findings of this study are associations between some clinical factors and vaccine acceptance and uptake among PwMDs. Because the rollout of COVID-19 vaccines is community-based in China, a significantly higher uptake rate in outpatients than in inpatients is expected. In the current study, persons with psychotic disorders were least likely to take the vaccine, which may be attributed to the severe impairment in decisional capacity of these people (36). For example, evidence from theory of mind studies shows that patients with schizophrenia significantly accept more disadvantageous offers and reject more advantageous offers (37). Overall, COVID-19 vaccination is beneficial for the health of patients with schizophrenia but with some uncertainties about the efficacy and safety; thus, these patients are more likely to delay or reject vaccination. PwMDs with good insight are more aware of the need for treatments to maintain their health, including vaccination; therefore, a significant positive association between good insight and vaccine acceptance is expected.

The results from the official surveillance report of adverse events of COVID-19 vaccines in China indicate that adverse reactions to COVID-19 vaccines are rare in Chinese residents and that most adverse reactions are normal reactions, such as fever and swelling (38). In this study, we investigated a broad range of adverse events by self-report. Although over one-fifth of the vaccine-recipients reported adverse events, these events were generally acceptable, suggesting the good safety of COVID-19 vaccines for PwMDs. Nevertheless, the 2.0% rate of exacerbated pre-existing psychiatric symptoms indicates that psychiatric follow-up is needed for PwMDs following the vaccination.

This study has several limitations. First, the study was conducted only during the early to middle period of the nationwide massive inoculation of vaccines; therefore, the current situations of vaccination in PwMDs during the late period of the massive inoculation of vaccines remain unclear. Second, our data on the safety of vaccines for PwMDs are preliminary given the small number of PwMDs who were vaccinated (*n* = 98). Third, this is a cross-sectional study; thus, the causal relationships between factors and vaccine uptake and acceptance need to be further examined in longitudinal studies. Fourth, a qualitative in-depth interview is useful for understanding the barriers to vaccination among PwMDs but we did not perform it. Finally, our assessment of psychiatric symptoms, based on self-report measures without verification from treating psychiatrists, might be subject to subjective bias. In addition, there might be social desirability bias in the results of assessment of PwMDs' attitudes toward COVID-19 vaccines.

In China, 17.5% of the adults suffer from a mental disorder during the prior month (39); therefore, to achieve the 80% COVID-19 herd immunity threshold, COVID-19 vaccination of PwMDs should not be neglected. In this study, ~60% of the PwMDs were willing to take the vaccine but only 10.8% of them were vaccinated. Our study revealed large discrepancies in the rates of vaccine acceptance and uptake among PwMDs and in the rates of COVID-19 vaccination coverage between PwMDs and the general population, which indicates that removing barriers to vaccination to increase PwMDs' vaccination coverage is an urgent task for both public health and mental health workers. Although our preliminary data show that vaccines are generally safe for PwMDs, the significant relationships between some clinical factors and vaccine uptake and acceptance suggest that the clinical characteristics of PwMDs should be considered in the development of targeted intervention strategies. PwMDs-specific strategies may include provision of reliable sources of information on vaccines, health education to improve their awareness of the efficacy and safety of vaccines, the development of a specialized guideline to facilitate primary care physicians' clinical decision-making for vaccination, joint vaccine consultation services that involve immunologists and psychiatrists, and psychosocial support and post-vaccination psychiatric follow-up services to prevent the relapse of mental disorders. In addition, more studies are warranted to recognize barriers to vaccination in PwMDs.

## Data Availability Statement

The raw data supporting the conclusions of this article will be made available by the authors, without undue reservation.

## Ethics Statement

The studies involving human participants were reviewed and approved by the Institutional Review Board of Wuhan Mental Health Center. The patients/participants provided their written informed consent to participate in this study.

## Author Contributions

B-LZ and HH designed the study, conducted the analyses, and wrote the initial version of this manuscript. HH, X-MZ, P-WL, Z-MF, WL, and Y-MM collected the sample and performed the literature review. X-MZ and HF-KC revised the manuscript. All authors edited, read, and approved the last version of this manuscript.

## Funding

This work was funded by the National Natural Science Foundation of China (Grant No. 81901351), the Natural Science Foundation of Hubei Province of China (Grant No. 2019CFB269), the Health Commission of Hubei Province Scientific Research Project (Grant No. WJ2019H352), and the Wuhan Municipal Health Commission Scientific Research Project (Grant No. WX19Q05).

## Conflict of Interest

The authors declare that the research was conducted in the absence of any commercial or financial relationships that could be construed as a potential conflict of interest.

## Publisher's Note

All claims expressed in this article are solely those of the authors and do not necessarily represent those of their affiliated organizations, or those of the publisher, the editors and the reviewers. Any product that may be evaluated in this article, or claim that may be made by its manufacturer, is not guaranteed or endorsed by the publisher.
